# Oral Health in Adults with Congenital Heart Disease

**DOI:** 10.3390/jcm8081255

**Published:** 2019-08-19

**Authors:** Matthias Folwaczny, Saskia Wilberg, Caspar Bumm, Stefan Hollatz, Renate Oberhoffer, Rhoia Clara Neidenbach, Harald Kaemmerer, Iris Frasheri

**Affiliations:** 1 Department of Conservative Dentistry and Periodontology, University Hospital, Ludwig-Maximilians-University, Goethestr. 70, D-80336 Munich, Germany; 2 Institute of Preventive Pediatrics, Department of Sport and Health Sciences, Technical University of Munich, D-80336 Munich, Germany; 3 German Heart Center Munich, Department of Congenital Heart Disease and Pediatric Cardiology, Technical University of Munich, D-80636 Munich, Germany

**Keywords:** bacteremia, infective endocarditis, periodontal, caries, odontogenic

## Abstract

Oral bacteria and odontogenic oral infections are responsible for a high portion of cases with infective endocarditis. Hence, oral health in patients with congenital heart disease (CHD) gains particular importance. This case-control study compared the oral health status in 112 adults with CHD and 168 healthy control subjects. In addition, the patient group was stratified according to the complexity of the heart defect and the recommendation for antibiotic prophylaxis during invasive dental procedures. Considering caries experience, a significantly lower mean DMFT (decayed missing filled teeth) score (7.91 ± 6.63 vs. 13.6 ± 8.15; *p* < 0.0001) was found in patients with CHD compared to healthy controls. Healthy controls had a higher average number of decayed teeth (0.33 ± 0.76 vs. 1.76 ± 2.61; *p* < 0.0001). In female subjects a significant lower relative amount of teeth with apical periodontitis was found among CHD patients (3.4% ± 0.9%) as compared to healthy controls (5.6% ± 1.9%) (*p* = 0.053). Regarding periodontal health, patients with CHD had lower rate of sulcus bleeding (0.32 ± 0.65 vs. 0.71 ± 0.60; *p* < 0.0001) and less alveolar bone loss than heart healthy individuals (% root length: multi rooted teeth: 8.97 ± 10.64 vs. 23.22 ± 20.70; *p* < 0.0001; single rooted teeth: 5.59 ± 6.25 vs. 17.30 ± 17.17; *p* = 0.003). On the contrary, CHD patients presented with higher amount of plaque in comparison to healthy controls (Quigley & Hein index: 2.22 ± 0.67 vs. 1.25 ± 0.72; *p* < 0.0001). Based on the current results, it can be concluded that adults with CHD have better oral health than heart healthy individuals.

## 1. Introduction

The diagnosis of congenital heart disease (CHD) encompasses various anatomical abnormalities of the heart and the great arteries [1]. Modern treatment methods are primarily directed towards the surgical repair of these structural defects. These treatment approaches are considerably life prolonging but mostly not curative, resulting in life-long persisting cardiovascular residua and sequels, e.g., residual shunts, valvular lesions, or implantation of prosthetic materials causing an inherent risk for infective endocarditis in these patients [2,3].

It is commonly accepted that the development of infective endocarditis requires bacteremia with microorganisms that can successfully adhere to the endocardial surfaces [4]. The oral cavity is colonized by a complex microflora and is frequently affected by two of the most prevalent human diseases, i.e., caries and periodontitis. Both are substantially caused by the manifestation of dysbiotic bacterial infections of tooth related structures [5]. Accordingly, the oral cavity is considered a highly relevant source for bacteremia leading to infective endocarditis. Bacteremia derived from the oral cavity is detectable after minor mucosal trauma, including not only various standard dental diagnostic and therapeutic procedures [6], but also frequent daily activities, i.e., teeth brushing or mastication [7]. Consistently, the risk for bacteremia with oral microorganisms is correlated with the presence and severity of oral diseases [8]. According to recent clinical observations, oral streptococci account for >20% of infective endocarditis [9].

Apart from the use of systemic antibiotics, the prophylaxis of transient bacteremia due to professional dental care and routine daily activities is directed towards the prevention and treatment of the oral disease itself. Despite the elevated risk of manifestation of infective endocarditis, there exists some evidence that young patients with CHD show increasing amounts of dental deposits, i.e., plaque, more plaque induced gingival inflammation, lower compliance with dental maintenance care, and poorer routine oral hygiene measures during childhood and adolescence [10,11]. In a study from Janssens, a high proportion of young patients with CHD aged 14–18 years did not have a dental visit during the past year and even more than 40% never flossed their teeth, indicating insufficient control of potentially infective bacterial deposits [12]. Yet, it remains to be elucidated if adults with CHD also show a lacking adherence to oral preventive measures ultimately resulting in a higher risk for caries, gingivitis or periodontitis. The present case-control study aimed to delineate (1) the level of oral care (2) the prevalence and severity of odontogenic diseases in adults with CHD in comparison to the general population.

## 2. Materials and Methods

### 2.1. Study Cohort

**Figure 1:**
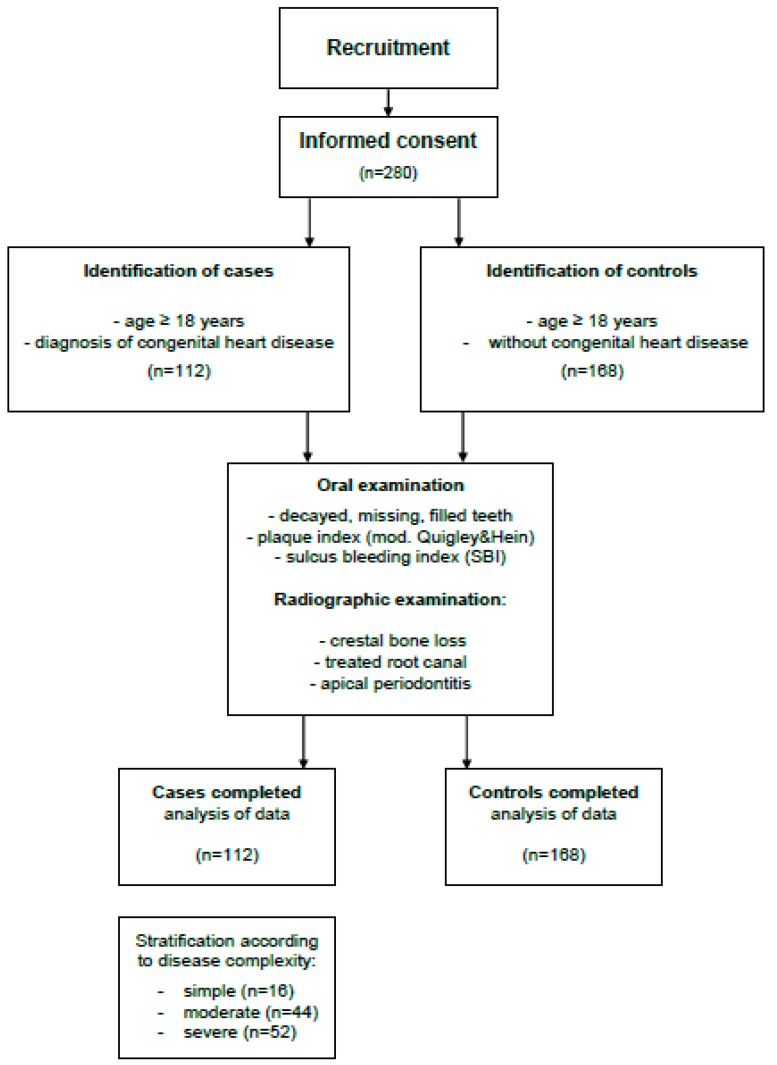
Flow diagram of recruitment process used for inclusion of cases and controls according to STROBE guidelines.

The patients were consecutively recruited among the out- and inpatients with CHD attending the German Heart Center Munich in July and August 2017. Individuals who were eligible for inclusion into the study cohort had to be aged 18 years or older and had a diagnosis of CHD. Each patient was assigned to one of three subgroups of CHD, according to their severity using the Bethesda criteria [13]. Controls were subjects without any known CHD attending the outpatient clinic of the Department of Restorative Dentistry and Periodontology of the University Hospital Munich for general dental care during August and December 2017 (Figure 1). Prior to enrollment into the study, all study subjects received detailed information about the objectives and methods of this study and gave their written informed consent. The study conformed to the ethical guidelines of the Helsinki Declaration and was approved by the Ethics Committees of the Medical Faculties of the Ludwig-Maximilians University (No. 17-708) and the Technical University Munich (No. 133/16S).

### 2.2. Clinical Examination of Patients

All study subjects received a standardized dental examination using mirror and probe as described previously [14]. Missing teeth, restorations, and untreated caries lesions were documented for each tooth in order to register the DMFT (decayed missing filled teeth) score for each study subject. Untreated caries lesions were registered according to the WHO criteria, which defines caries as lesions within the tooth surface and/or secondary caries at restorations or prosthetic crowns. In order to avoid study-related bacteremia, the clinical periodontal examination of study subjects was confined to the determination of the sulcus bleeding index (SBI) according to Mühlemann et al. [15], evaluating the clinical appearance and the tendency of gingival bleeding as surrogate for the manifestation of gingivitis. The presence of plaque at unrestored facial and lingual tooth surfaces has been quantitatively assessed using the modified Quigley Hein index [16].

### 2.3. Radiographic Examination

Radiographic examination was performed in CHD-patients and control subjects using panoramic X-ray and apical radiographs as made during regular dental care. Due to ethical reasons appropriate X-rays were available only when radiographic examination was medically indicated. Radiographic findings regarding the periodontal status of study subjects was determined as the maximum amount of alveolar bone loss, indicated as a percentage of the root length, using the radiographic apex apically and a line 1 mm apical from the dento-enamel junction as crestal reference. Complementary evidence regarding carious defects, specifically approximal caries, was documented. In addition, radiographic signs, i.e., radiolucent areas indicating apical periodontitis, and previous root canal treatment was recorded for each patient.

### 2.4. Statistical Analysis

Sample size calculation was performed under the assumption of an effect size d = 0.51 based on the mean DMFT scores for cases and controls as found in the age group 8–12 years for permanent teeth by Ali et al. [11] using the G-Power Calculator (version 3.1). To reach a power of 0.9 with an allocation ratio of 1.5 between controls and cases, a minimum of 180 study subjects (72 with CHD and 108 healthy controls) had to be enrolled. For all continuous variables, mean and standard deviation were calculated. Distribution of data within the two study groups was tested for normal distribution using the Kolmogorov-Smirnov procedure. Since data were not normally distributed, univariate analysis of differences between study groups was performed with a Kruskal–Wallis and Mann–Whitney test where appropriate. In addition, a subgroup analysis according to gender was made. For correlation of clinical and radiographic findings among patients with CHD with the complexity of the disease, the main CHD diagnosis and the recommendation for antibiotic prophylaxis prior to invasive dental treatment the study group was stratified. Due to the high variation of individual diagnoses among patients, CHD diagnosis was included into the analysis only, if ≥10 patients provided the same diagnosis. For categorical data the Fisher’s exact test was applied. All data are shown as mean values (± SD) or relative frequencies. Cases with missing values were removed from the analysis. To determine the impact of CHD on caries, periodontitis, and oral hygiene measures, a binary logistic regression analysis was performed using congenital heart disease, age and gender as independent factors. Odds ratios (OR) and 95% confidence intervals and the effect size f according to Cohen et al. [17] were calculated. For logistic regression analysis, the dependent variables were transformed into dichotomous categories, i.e., study subjects were categorized according to the absence or presence of (1) previously decayed, missing or filled teeth (DMFT score 0 vs. >0), (2) actual unrestored caries lesion, (3) previous root canal treatment and/or apical periodontitis, (4) periodontal bone loss (bone loss <10% vs. ≥10% of total root length), and (5) supragingival plaque (QH score ≤1 or >1). *p* values < 0.05 were considered significant. All test procedures were two-tailed and performed using the SPSS Software Program (version 23, SPSS Inc., Chicago, IL, USA).

## 3. Results

### 3.1. Study Sample

The study group comprised a total of 112 patients (56 males, 56 females) with CHD, among which the complexity of the CHD was classified as simple in 16 patients (14.3%), moderate in 44 patients (39.3%), and as severe in 52 patients (46.4%). The mean age in the patient group was 34.4 (±12.6) years. A total of 168 patients (87 male, 81 female) with a mean age of 43.1 (±18.9) years were enrolled into the control group. The age was significantly different between groups (*p* < 0.0001), whereas the male to female ratio was not different (*p* = 0.432) (Table 1). Considering the frequency distribution of single diagnosis among CHD patients, 24 subjects had transposition of great arteries and 12 patients were diagnosed as having tetralogy of Fallot (Table 2).

### 3.2. Caries Experience

Patients with CHD presented with a mean DMFT score of 7.91 (±6.63) compared to 13.6 (±8.15) in the control group (*p* < 0.0001). In line, also the average number of decayed teeth was higher among healthy control individuals (1.76 ± 2.61) than for patients with CHD (0.33 ± 0.76) (*p* < 0.0001) (Table 3). Logistic regression analysis revealed a decreased chance for patients with CHD to present with active carious lesions (OR: 0.20; 95% CI: 0.11–0.36; *p* < 0.0001), indicating a strong effect size (effect size f: 0.53) (Table 4). Following stratification of the study group according to the gender the DMFT and the number of decayed teeth remained significantly higher in healthy individuals than in patients with CHD when a separate analysis for male and female subjects was performed.

### 3.3. Endodontic and Periapical Status

Radiographic evaluation revealed that a mean of 8.8% (±5.8%) teeth received previous root canal treatment among patients with CHD as compared to 8.6% (±4.7%) teeth in healthy controls (*p* = 0.89). Moreover, 3.4% (±0.7%) of teeth presented with a diagnosis of apical periodontitis in patients with heart defects and 4.8% (±2.1%) of the teeth in healthy individuals (*p* = 0.129) (Table 5). After stratification of the study sample according to the gender, female subjects with CHD had a significant smaller number of teeth with apical periodontitis than healthy control females (*p* = 0.053). Discerning the patients with CHD according to the recommendation for antibiotic prophylaxis, a higher average number of teeth with periapical periodontitis was found among patients who needed antibiotic prophylaxis than patients without recommendation, although not reaching significance.

### 3.4. Gingival and Periodontal Status

The mean sulcus bleeding index (SBI) which reflects the tendency for gingival bleeding, was 0.32 (±0.65) for patients with CHD and 0.70 (±0.60) for healthy controls (*p* = 0.001) (Table 6). The manifestation of supragingival plaque was higher in patients (2.22 ± 0.0.67) compared to the healthy control individuals (1.25 ± 0.72) (*p* < 0.0001), as represented by the Quigley and Hein index score. Considering regression analysis, patients with CHD presented with a considerably higher chance for the manifestation of plaque (OR: 9.72; 95% CI: 3.66–25.82; *p* < 0.0001) (Table 4). The comparison of the maximum alveolar bone loss at both, single rooted and multi rooted teeth, revealed a significant higher bone loss for controls than for patients (*p* < 0.0001) (Table 7). Consistently, the odds for patients with CHD showing periodontal bone loss were smaller as compared to healthy controls, but did not reach significance (OR: 0.48; 95% CI: 0.17–1.32; *p* = 0.155).

## 4. Discussion

The individual susceptibility for the manifestation of infective endocarditis is strongly influenced by the presence and severity of oral diseases [7,18]. The manipulation and perforation of infected tissue, particularly the gingival margin and the periapical tissue, during dental care and routine daily activities, i.e., teeth brushing or mastication, causes an inherent risk for bacteremia [19]. Antibiotic prophylaxis has been recommended to address the elevated risk for infective endocarditis during occasional diagnostic and therapeutic dental procedures in patients with cardiovascular anomalies, including individuals with CHD [20,21]. Considering daily activities, the avoidance of oral diseases seems the most appropriate prevention strategy against infective endocarditis in individuals with predisposing cardiovascular defects [22]. Comparing the experience with caries and periodontitis between individuals with and without CHD, patients with heart disease presented a considerably better oral health. Focusing on caries, the DMFT reflects the number of already missing, previously restored, and actually decayed teeth. This score was significantly lower among patients with CHD than without. As already reported, these patients with CHD had an average DMFT index of 7.91 [14]. In the control group examined in the present study, the DMFT reached even 13.59, indicating a considerably higher caries experience for heart-healthy individuals. This might be explained by a stricter adherence to oral maintenance care by individuals with CHD. Thereby, the current observations confirm the results of a recent Swedish study, which found a considerably lower rate of new approximal caries lesions in adolescents with CHD than in healthy controls [23]. The lower incidence of caries in individuals with heart defects was specifically referred to their enrollment into a systematic preventive program already at the age of one year. However, considering the differences in caries experience between CHD patients and healthy controls one has to bear in mind the higher mean age within the latter group. The DMFT shows an age depending continuous increase. The increase of the DMFT was 3.7, comparing individuals at an age of 30 and 40 years according to a previous survey [24]. The differences as observed herein might, therefore, at least partially attributable to the higher mean age of healthy controls.

Despite the overall lower caries experience among individuals with CHD, the subgroup of patients needing antibiotic prophylaxis prior to invasive dental procedures, however, showed significantly elevated DMFT scores as previously reported [14], compared to the remaining cohort. Although not reaching significance this result is in line with the observation of a higher DMFT among patients with TGA as compared to subjects with tetralogy of Fallot representing moderate and severe forms of CHD respectively. Since the recommendation for antibiotic prophylaxis is particularly related to patients with severe forms of CHD due to their extraordinary high risk for infective endocarditis [19], this observation seems alarming. It might be explained by stronger concerns about the risk to induce bacteremia during regular oral hygiene measures among patients with more severe forms of CHD.

Progressing caries finally reaches the pulp chamber and causes necrosis of the pulp tissue, ultimately resulting in bacterial infection and inflammation of the periapical tissue. Root canal treatment is the commonly accepted therapeutic approach to address both entities. Despite no clear evidence, teeth showing periapical disease have been suggested to cause transient bacteremia in remote sites of the organism [20,24]. Herein, heart healthy subjects presented a higher average number of teeth with periapical disease than patients with CHD. However, this difference between the groups was significant in female subjects only. One might speculate that female patients with CHD adhere better to oral supportive care and are more rigorous in maintaining oral health than male patients. Since periapical periodontitis is a consequence of progressing caries, again, these data seem to further support the conclusion that patients with CHD have a lower caries experience than healthy controls.

The total soft tissue surface area of periodontal pockets amounts to almost 44 cm^2^ [25] and has thus been proposed as a highly significant source of entry for bacteria invading the systemic bloodstream [26]. The surface area seems to correlate with the progression of periodontitis due to the deepening of the periodontal pockets [25]. Based on radiographic data, periodontal bone loss at both single rooted and multi rooted teeth was three times as high in heart healthy individuals compared to subjects with CHD. Although the difference did not reach significance, there was, again, a trend for a more pronounced bone loss among patients with CHD, for whom antibiotic prophylaxis is recommended prior to invasive dental procedures. The current observations are partially contradictory to two previous studies in children revealing a higher rate of gingivitis, which is commonly considered as precursor disease to periodontitis, among patients with CHD [10,11].

It is commonly accepted that the manifestation and progression of periodontitis and caries is essentially bound to the presence of plaque [5]. Intriguingly, despite their considerably lower experience regarding caries and periodontitis, patients with CHD presented with significantly higher amounts of plaque compared to heart-healthy controls herein. This might lead to the assumption of poorer compliance with oral hygiene measures. As previously reported, an attitude of neglecting the clinical relevance of regular oral hygiene among the CHD cohort of this study was found [14]. The higher amount of plaque found in patients with CHD might also be explained by differences in the clinical conditions under which it has been determined in both study groups. Healthy control patients attended a university dental school and were thus aware about the examination and/or treatment of their teeth. This might have motivated them to tooth cleaning measures immediately prior to their visit. On the contrary, patients with CHD were examined during routine cardiological follow-up visits at the cardiological clinic, being unaware of the dental examination in this context. This conclusion is supported by the lower rate of sulcus bleeding among CHD patients than in healthy controls. It is commonly accepted that gingival bleeding correlates with the amount of plaque during the past days prior to the examination. Gingival bleeding in the absence of actual plaque confirms plaque that has just been removed immediately prior to the clinical determination of the bleeding score. On the contrary, the absence of bleeding together with the manifestation of current plaque is compatible with effective plaque control within the past days.

The current data reveal distinct differences of oral health between individuals with CHD and heart healthy controls. When drawing conclusions, at least two specific aspects of the study design have to be taken into account. Most important, healthy control subjects were recruited in a tertiary dental hospital center, probably leading to selection bias. This problem was addressed by the random selection of controls among patients attending the hospital for dental treatment by undergraduate students. These patients typically present with common dental problems and appear as a representative sample of the general population. Moreover, since the oral examination was performed at two different hospital centers, clinical data were not collected by the same person in each patient, potentially resulting in a certain degree of interexaminer variability.

## 5. Conclusions

Taken together, the findings of this study show a considerably lower experience of caries and periodontitis among adults with CHD. Subgroup analysis however revealed significantly higher DMFT scores and more radiographic bone loss in subjects with an indication for antibiotic prophylaxis prior to invasive dental treatment, who are exposed to a particularly elevated risk of infective endocarditis.

## Figures and Tables

**Table 1:** Of study groups; *p*-values as obtained with Mann–Whitney and Fisher’s exact test; complexity of CHD according to Bethesda criteria.

	CHD	Control	*p*-Value
number of cases	*n* = 112	*n* = 168	
age (years ± SD)	34.5 (± 12.6)	43.1 (± 18.9)	<0.0001
gender			
(male/female)	5%/50%	51.8%/48.2%	0.432
CHD complexity			
simple	*n* = 16 (14.3%)		
moderate	*n* = 44 (39.3%)		
severe	*n* = 52 (46.4%)		

CHD: congenital heart disease.

**Table 2:** With caries among patients with CHD and healthy control subjects. Analysis of differences between groups has been done with Mann–Whitney test, analysis between subgroups (CHD complexity) with Kruskal–Wallis test.

	DMFT (*n*)	Decayed Teeth (*n*)
**total**		
CHD group	7.91 (±6.63)	0.33 (±0.76)
cases analyzed	112	112
control group	13.60 (±8.15)	1.76 (±2.61)
cases analyzed	168	168
*p*-value	<0.0001	<0.0001
**female**		
CHD group	8.77 (±6.99)	0.32 (±0.79)
control group	13.77 (±8.87)	1.30 (±1.89)
*p*-value	0.001	<0.0001
**male**		
CHD group	7.05 (±6.2)	0.34 (±0.75)
control group	13.44 (±7.47)	2.20 (±3.09)
*p*-value	<0.0001	<0.0001
**CHD complexity**		
simple	9.38 (±8.04)	0.13 (±0.50)
moderate	7.39 (±7.20)	0.27 (±0.69)
severe	7.90 (±5.68)	0.44 (±0.87)
*p*-value	0.449	0.189
**antibiotic prophylaxis**		
yes	9.96 (±6.81)	0.50 (±0.98)
no	6.13 (±5.98)	0.18 (±0.47)
*p*-value	0.001	0.086

DMFT: decayed missing filled teeth.

**Table 3:** Experience with caries among patients with CHD and healthy control subjects. Analysis of differences between groups has been done with Mann–Whitney test, analysis between subgroups (CHD complexity) with Kruskal–Wallis test.

	DMFT (*n*)	Decayed Teeth (*n*)
**total**		
CHD group	7.91 (±6.63)	0.33 (±0.76)
cases analyzed	112	112
control group	13.60 (±8.15)	1.76 (±2.61)
cases analyzed	168	168
*p*-value	<0.0001	<0.0001
**female**		
CHD group	8.77 (±6.99)	0.32 (±0.79)
control group	13.77 (±8.87)	1.30 (±1.89)
*p*-value	0.001	<0.0001
**male**		
CHD group	7.05 (±6.2)	0.34 (±0.75)
control group	13.44 (±7.47)	2.20 (±3.09)
*p*-value	<0.0001	<0.0001
**CHD complexity**		
simple	9.38 (±8.04)	0.13 (±0.50)
moderate	7.39 (±7.20)	0.27 (±0.69)
severe	7.90 (±5.68)	0.44 (±0.87)
*p*-value	0.449	0.189
**diagnosis CHD**		
Fallot	5.42 (±5.93)	0.08 (±0.29)
*p*-value (vs. control)	0.001	0.001
TGA	7.50 (±6.47)	0.42 (±0.83)
*p*-value (vs. control)	0.001	0.002
*p*-value (Fallot vs. TGA)	0.267	0.215
**antibiotic prophylaxis**		
yes	9.96 (±6.81)	0.50 (±0.98)
no	6.13 (±5.98)	0.18 (±0.47)
*p*-value	0.001	0.086

DMFT: decayed missing filled teeth, Fallot: tetralogy of Fallot, TGA: transposition of great arteries.

**Table 4:** Results of binary logistic regression analysis using previous caries experience, present unrestored carious lesions, previous root canal treatment and/or apical periodontitis, supragingival plaque and periodontal bone loss as dependent variables. Significance of regression coefficient B has been tested with Wald test, results are presented as *p*-values. The effect size f has been calculated with Nagelkerkes R-squared.

	OR (95% CI)	Regression Coefficient B	*p*-Value	Effect Size f
**caries experience (DMFT score 0 vs. >0)**				
gender	0.88 (0.32–2.46)	–0.123	0.813	0.38
age	1.11 (1.04–1.19)	0.105	0.003	
heart disease	0.77 (0.28–2.12)	–0.265	0.609	
**present carious lesion (absence vs. presence)**				
gender	0.60 (0.36–1.01)	–0.516	0.052	0.53
age	1.02 (1.00–1.03)	0.016	0.038	
heart disease	0.20 (0.11–0.36)	–1.604	<0.0001	
**RCT/apical periodontitis (absence vs. presence)**				
gender	0.73 (0.31–1.71)	–0.312	0.470	0.47
age	1.04 (1.01–1.07)	0.039	0.007	
heart disease	0.58 (0.23–1.51)	–0.537	0.226	
**supragingival plaque (QH score ≤1 vs. >1)**				
gender	0.74 (0.38–1.43)	–0.298	0.375	0.49
age	1.03 (1.01–1.05)	0.027	0.009	
heart disease	9.72 (3.66–25.82)	2.274	<0.0001	
**periodontal bone loss (<10% vs. ≥10% root length)**				
gender	1.10 (0.41–2.93)	0.091	0.856	0.87
age	1.09 (1.05–1.14)	0.086	<0.0001	
heart disease	0.48 (0.17–1.32)	–0.737	0.155	

OR: odds ratio; CI: confidence interval, DMFT: decayed missing filled teeth; RCT: previous root canal treatment; QH: Quigley and Hein index.

**Table 5:** Amount of teeth with previous root canal treatment and apical periodontitis among patients with CHD and healthy control subjects. Analysis of differences between groups has been done with Mann–Whitney test, analysis between subgroups (CHD complexity) with Kruskal–Wallis test.

	Teeth with RCT (%)	Teeth with AP (%)
**total**		
CHD group	8.8 (±5.8)	3.4 (±0.7)
cases analyzed	34	34
control group	8.6 (±4.7)	4.8 (±2.1)
cases analyzed	76	76
*p*-value	0.890	0.129
**female**		
CHD group	6.9 (±1.9)	3.4 (±0.9)
control group	9.2 (±4.6)	5.6 (±1.9)
*p*-value	0.290	0.053
**male**		
CHD group	9.8 (±7.0)	3.6 (±0.1)
control group	8.2 (±4.8)	4.3 (±2.2)
*p*-value	0.414	0.655
**CHD complexity**		
simple	7.6 (±0.6)	4.9 (n.d.)
moderate	9.2 (±8.0)	3.6 (n.d.)
severe	8.7 (±5.8)	3.2 (±0.8)
I-value	0.949	0.712
**antibiotic prophylaxis**		
yes	8.7 (±4.2)	3.7 (±0.1)
no	8.8 (±7.4)	3.2 (±1.0)
*p*-value	0.962	455

RCT: root canal treatment; AP: apical periodontitis.

**Table 6:** Oral health parameters among patients with congenital heart disease and healthy control subjects. Analysis of differences between groups has been done with Mann–Whitney test, analysis between subgroups (CHD complexity) with Kruskal-Wallis test.

	Quigley & Hein (Average)	Quigley & Hein (Maximum)	SBI (Average)	SBI (Maximum)
**total**				
CHD group	2.22 (±0.67)	2.89 (±0.85)	0.32 (±0.65)	0.94(±1.15)
cases analyzed	111	111	35	35
control group	1.25 (±0.72)	2.08 (±0.97)	0.71 (±0.60)	1.32 (±0.83)
cases analyzed	166	166	162	162
*p*-value	<0.0001	<0.0001	<0.0001	0.002
**female**				
CHD group	1.99 (±0.71)	2.67 (±0.90)	0.23 (±0.30)	0.88 (±1.17)
control group	1.14 (±0.63)	1.97 (±0.91)	0.58 (±0.56)	1.13 (±0.85)
*p*-value	<0.0001	<0.0001	0.008	0.106
**male**				
CHD group	2.44 (±0.55)	3.11 (±0.73)	0.43 (±0.89)	1.00 (±1.17)
control group	1.35 (±0.78)	2.17 (±1.01)	0.82 (±0.62)	1.49 (±0.78)
*p*-value	<0.0001	<0.0001	<0.0001	0.009
**CHD complexity**				
simple	2.06 (±0.57)	2.73 (±0.70)	0.00 (±0.00)	0.00 (±0.00)
moderate	2.09 (±0.65)	2.82 (±0.87)	0.50 (±1.09)	1.18 (±1.25)
severe	2.37 (±0.69)	3.00 (±0.86)	0.31 (±0.31)	1.06 (±1.16)
*p*-value	0.096	0.238	0.034	0.044
**antibiotic prophylaxis**				
yes	2.28 (±0.71)	2.92 (±0.82)	0.38 (±0.82)	0.95 (±1.10)
no	2.16 (±0.63)	2.87 (±0.87)	0.24 (±0.34)	0.93 (±1.27)
*p*-value	0.358	0.706	0.564	0.769

QH: Quigley and Hein index; SBI: sulcus bleeding index.

**Table 7:** Periodontitis among patients with CHD and healthy control subjects. Analysis of differences between groups has been done with Mann–Whitney test, analysis between subgroups (CHD complexity) with Kruskal–Wallis test. Bone loss: loss of supporting bone in % of root length.

	Bone Loss Multi Rooted (% Root Length)	Bone Loss Single Rooted (% Root Length)
**total**		
CHD group	8.97 (±10.64)	5.59 (±6.25)
cases analyzed	34	34
control group	23.22 (±20.70)	17.30 (±17.17)
cases analyzed	76	76
*p*-value	<0.0001	0.003
**female**		
CHD group	10.0 (±13.46)	6.47 (±6.32)
control group	26.41 (±22.4)	20.16 (±18.30)
*p*-value	0.009	0.015
**male**		
CHD group	7.94 (±7.08)	4.71 (±6.24)
control group	20.91 (±19.27)	15.23 (±16.02)
*p*-value	0.015	0.045
**CHD complexity**		
simple	10.00 (±10.00)	6.00 (±6.52)
moderate	4.67 (±5.16)	3.33 (±4.50)
severe	13.21 (±13.67)	7.86 (±7.26)
*p*-value	0.110	0.192
**antibiotic prophylaxis**		
yes	10.00 (±8.37)	6.25 (±6.19)
no	8.06 (±12.50)	5.00 (±6.42)
*p*-value	0.154	0.463
